# Maternal and Neonatal Determinants of Respiratory Outcome Following Second-Trimester PPROM: A Multi-Domain Machine Learning Analysis

**DOI:** 10.3390/diagnostics16121911

**Published:** 2026-06-19

**Authors:** Simon Loth, Julia Hauer, Christoph Scholz, Marcus Krüger, Alexander Bieber, Christian Brickmann

**Affiliations:** 1 Department of Pediatrics, TUM School of Medicine and Health, Kinderklinik Muenchen Schwabing, TUM University Hospital, Technical University of Munich, 80804 Munich, Germany; 2 Department of Neonatology, TUM School of Medicine and Health, TUM University Hospital, Technical University of Munich, 80804 Munich, Germany; 3 Clinic for Gynaecology and Obstetrics, Muenchen Klinik Harlaching gGmbH, 81545 Munich, Germany; 4 Clinic for Neonatology, Muenchen Klinik, 81545 Munich, Germany

**Keywords:** preterm premature rupture of membranes, machine learning, pulmonary hypoplasia, amniotic fluid trajectory, neonatal risk stratification

## Abstract

**Background**: Preterm premature rupture of membranes (PPROM) before 32 weeks of gestation with prolonged latency is associated with substantial neonatal morbidity, including Dry Lung Syndrome (DLS), pulmonary hypoplasia (PH), bronchopulmonary dysplasia (BPD), and death. Accurate individualized risk stratification remains elusive, as the interacting contributions of amniotic fluid dynamics, inflammatory status, and microbiological burden are inadequately captured by traditional statistical approaches. **Methods**: We performed a retrospective, exploratory–predictive analysis of 66 pregnancies complicated by second-trimester PPROM with latency exceeding 14 days. Elastic Net and Random Forest models were trained across six clinically defined predictor domains using a multi-stage block modelling strategy. To address the clinically relevant distinction between antenatal and postnatal information, results are reported separately for Model A—comprising exclusively antenatal predictors available during expectant management (gestational age at PPROM, latency, amniotic fluid trajectory, inflammatory status, vaginal microbiome at admission)—and Model B, which additionally incorporates postnatal variables and characterizes the full mechanistic perinatal risk trajectory. Binary and ordinal outcomes included DLS, PH, BPD, intraventricular hemorrhage (IVH), and neonatal death. Pairwise interaction models were additionally computed to identify cross-domain risk constellations. **Results**: Distinct predictor architectures emerged per outcome. Pulmonary hypoplasia was most strongly associated with temporal features of oligohydramnios—particularly the persistence and timing of SDP < 1 cm—rather than isolated measurements. For DLS, the antenatal model (Model A) achieved AUC 0.776, driven by gestational maturity and inflammatory status; surfactant administration—a postnatal variable reflecting therapeutic response rather than an antenatal risk factor—dominated only the mechanistic Model B. Neonatal death was driven by a combined profile of respiratory support burden, amniotic fluid persistence, and co-morbidity. IVH showed consistently high ordinal predictability (accuracy 0.863), with amniotic fluid dynamics and microbiological burden as leading contributors. BPD remained the least linearly separable endpoint across all configurations. **Conclusions**: Multi-domain machine learning reveals outcome-specific, cross-domain risk architectures following second-trimester PPROM that are invisible to conventional statistical models. Longitudinal amniotic fluid trajectory is the dominant antenatal determinant of structural pulmonary morbidity, while microbiological burden independently shapes neurological risk. These findings support prospective validation of integrated ML-based risk stratification tools for individualized antenatal counselling in this high-risk population.

## 1. Introduction

Preterm premature rupture of membranes (PPROM) complicates approximately 2–5% of all pregnancies and accounts for approximately one third of spontaneous preterm births [1,2,3,4,5]. When PPROM occurs before 32 weeks of gestation with a prolonged latency period exceeding 14 days, clinical management becomes particularly challenging: the competing risks of extreme prematurity, prolonged oligohydramnios, ascending infection, and impaired pulmonary development must be balanced against the potential benefit of further uterine maturation [6,7,8,9]. Despite improvements in perinatal care, predicting which individual neonate will develop severe complications—in particular dry lung syndrome (DLS), pulmonary hypoplasia (PH), or bronchopulmonary dysplasia (BPD)—remains elusive, and reliable tools for individualized antenatal risk stratification are lacking [10].

Neonatal respiratory outcomes in this population span a wide spectrum. At the severe end, PH—arising from prolonged restriction of fetal lung development during the canalicular phase—is associated with high early mortality and persistent respiratory failure [7,11,12,13]. At the milder end, DLS represents a transient functional airway collapse following loss of lung fluid, characterized by severe immediate respiratory distress that resolves rapidly with high inflation pressures, in contrast to the irreversible structural changes in true hypoplasia [14,15,16]. Distinguishing between these two phenotypes antenatally remains a major diagnostic challenge [10]. Both conditions are closely linked to the degree and duration of oligohydramnios, with the gestational age at membrane rupture and the trajectory of amniotic fluid volume throughout the latency period representing critical prognostic determinants [4,11,17,18].

Maternal inflammatory status represents a further key dimension of risk. Intra-amniotic inflammation—present in approximately 40% of PPROM cases—is driven by microbial invasion of the amniotic cavity and mediated by pro-inflammatory cytokines including IL-6, IL-1β, and inflammasome activation markers such as ASC [19]. Whether of infectious or sterile origin, intra-amniotic inflammation is associated with adverse neonatal outcomes and contributes independently to the risk of early-onset sepsis, BPD, and perinatal death [19,20,21,22]. Importantly, the vaginal microbiome constitutes a critical upstream modulator of this inflammatory cascade. In healthy pregnancy, Lactobacillus dominance maintains a protective low-pH environment; its depletion, and the emergence of dysbiotic communities rich in Gardnerella, Ureaplasma, and Prevotella species, is associated with elevated matrix metalloproteinases, membrane weakening, and ascending infection. Antibiotic treatment during expectant management further perturbs microbial community structure, with potential implications for neonatal sepsis risk [23,24,25]. Monitoring vaginal microbiome dynamics—from PPROM diagnosis through delivery—may, therefore, provide prognostically relevant information beyond static maternal inflammatory markers.

Traditional statistical approaches have relied on logistic regression with a limited set of baseline variables, offering insights into individual risk factors but capturing neither the complex, nonlinear interactions among multiple concurrent exposures nor the longitudinal evolution of clinical parameters over the latency period [26]. Machine learning (ML) methods offer a conceptually more appropriate framework for this clinical problem. Algorithms including random forests, gradient boosting, and neural network architectures can model high-dimensional interactions without pre-specified functional forms and are particularly suited to integrating trajectorial data—such as serial amniotic fluid measurements—alongside static clinical, inflammatory, and microbiome variables [26,27,28,29]. Interpretability tools such as SHAP values further allow clinically meaningful decomposition of individual-level predictions, supporting translational application in counselling and decision-making [27,30].

Our group has previously described the respiratory outcome spectrum following prolonged second-trimester PPROM using a multifactorial longitudinal approach, demonstrating that gestational age at rupture, amniotic fluid trajectories, and inflammatory and microbiome status interact to shape neonatal pulmonary phenotype [10]. The present study extends this work by systematically integrating these data domains within a machine learning framework to develop robust predictive models for short-term neonatal outcomes—with particular focus on DLS, PH, and mortality—in a cohort of newborns exposed to PPROM before 32 weeks with latency periods exceeding 14 days. By systematically separating antenatal predictor domains—available to the clinician during expectant management—from postnatal treatment variables that reflect the disease course itself, we aim to identify the true antenatal prognostic signal, characterize the full mechanistic risk architecture, and provide a structured basis for evidence-based individualized counselling in this high-risk population.

## 2. Methods

### 2.1. Study Design and Data Source

This study is based on a retrospective, exploratory-predictive evaluation of a clinical dataset comprising pregnancies complicated by preterm premature rupture of membranes (PPROM). The primary aim was to identify relevant predictors of neonatal complications following PPROM using a multi-domain approach, and to systematically compare the predictive performance of individual variable blocks as well as combined overall models. Analyses were conducted at the level of individual clinical cases, with one complete case record per observation unit.

The dataset encompassed baseline clinical information, inflammatory laboratory parameters, serial sonographic amniotic fluid measurements, microbiological findings, and data on neonatal care and respiratory support. Statistical analyses were performed using R (version 4.5.2). The following packages were employed for data processing and modelling: *readxl*, *dplyr*, *tidyr*, *stringr*, *purrr*, *caret*, *glmnet*, *ranger*, *pROC*, *tibble*, and *Matrix*. A fixed random seed (set.seed = 123) was applied to ensure reproducibility.

### 2.2. Data Preparation and Preprocessing

Following data import, all variables underwent structured preprocessing. Automated type detection was applied to distinguish numeric from categorical variables. Variables with numeric content were standardized and converted to numeric format for further processing.

Categorical variables were encoded as factor variables. Empty entries and missing values in categorical variables were assigned to an explicit missing category, thereby retaining incomplete observations in the analytical pipeline. Missing values in numeric variables were imputed with the median of the respective variable prior to modelling; where no median could be determined due to entirely absent values, a replacement value of zero was applied. This approach ensured robust inclusion of partially incomplete predictors in the subsequent machine learning procedures.

Prior to modelling, variables with insufficient variance were excluded. All predictors were assessed for the presence of more than one non-missing value; variables without meaningful dispersion were removed, as they could not contribute discriminative information to model construction.

### 2.3. Descriptive Analysis and Missingness

In addition to predictive modelling, a comprehensive descriptive characterization of the dataset was performed. For each variable, the number of observations, absolute and relative counts of missing values, and—depending on data type—measures of central tendency and dispersion were calculated. For numeric variables, these comprised mean, standard deviation, median, interquartile range, minimum, and maximum. For categorical variables, the most frequently observed category was determined. A dedicated missingness table was compiled, documenting the absolute and relative frequency of missing values for each variable. This descriptive pre-analysis served both to assess the quality of the data and to contextualize the subsequent modelling results.

### 2.4. Outcome Definition

Several clinically relevant neonatal endpoints were defined as primary outcomes. Binary outcomes included neonatal death, dry lung syndrome (DLS), pulmonary hypoplasia, bronchopulmonary dysplasia (BPD), and intraventricular hemorrhage (IVH). Binary encoding followed a rule-based procedure: numeric values greater than zero were coded as events, values of zero as non-events. For non-numeric outcome variables, commonly used text-based codes (e.g., “*yes*”, “*ja*”, “*positive*” vs. “*no*”, “*nein*”, “*negative*”) were converted to binary event indicators. The resulting binary outcome variables were modelled as factors with levels “*yes*” and “*no*”.

Pulmonary outcomes were classified into three mutually exclusive postnatal respiratory phenotypes: no respiratory distress, DLS, and PH. DLS was defined as severe initial respiratory failure following prolonged oligohydramnios requiring invasive mechanical ventilation, with rapid clinical improvement and successful weaning within 48 h, consistent with the functional airway collapse phenotype described [14,15,16]. PH was defined as persistent postnatal respiratory failure despite standard management for prematurity-related RDS, requiring invasive mechanical ventilation beyond 48 h, characterized by sustained high ventilatory and oxygen requirements, limited radiographic lung expansion with low lung volumes, and/or associated pulmonary hypertension where documented. Duration of invasive ventilation (≤48 h vs. >48 h) served as the operational criterion distinguishing the two phenotypes.

For BPD and IVH, the presence of more than two ordered severity levels within the dataset was additionally assessed. Where applicable, ordinal outcome variants were generated to allow graded clinical severity to be incorporated alongside the binary event structure. Where a variable was predominantly numerically coded, ordinal factor levels were assigned based on numeric values; otherwise, ordering was derived from categorical levels.

### 2.5. Definition of Predictor Domains

To systematically represent the complexity of clinical information, predictors were organized into several content-defined variable blocks. This block structure formed the basis of the multi-stage modelling strategy.

*Base block*: comprised maternal and perinatal baseline characteristics, including gestational age at membrane rupture, latency from rupture to delivery, gestational age at birth, neonatal sex, and mode of delivery.

*Inflammation block*: included clinically established inflammatory parameters, specifically the presence of an inflammatory-infectious syndrome, and maximum values of C-reactive protein (CRP) and leucocyte count.

*Neonatal care block*: encompassed early postnatal therapeutic measures, particularly antenatal corticosteroid administration and surfactant therapy.

*Ventilation block*: captured the duration of invasive mechanical ventilation and non-invasive ventilatory support.

*Amniotic fluid dynamics block*: represented the trajectory of amniotic fluid volume based on serial single deepest pocket (SDP) measurements.

*Microbiology block*: comprised vaginal microbiological findings at the time of PPROM and at delivery. For the purposes of antenatal risk stratification, the base, inflammation, amniotic fluid dynamics, and microbiology at admission blocks were classified as antenatal predictor domains (Model A), representing information available during expectant management. The neonatal care block (including surfactant administration) and ventilation block represent postnatal or peripartum information and were designated as Model B predictors, characterizing the mechanistic risk trajectory. This distinction is maintained explicitly throughout the reporting of results.

This domain-specific structure allowed both the isolated predictive relevance of individual clinical levels and their incremental contribution within combined models to be evaluated.

### 2.6. Derivation of Longitudinal Amniotic Fluid Features

A central methodological focus of the analysis was the detailed representation of sonographically assessed amniotic fluid trajectories derived from serial SDP measurements. Beyond raw variables such as minimum amniotic fluid volume and median SDP, additional trajectory features were generated from time-stamped measurements obtained at admission and in subsequent weeks. The aim was to capture not only static values but also the dynamics, persistence, and temporal patterns of the amniotic fluid situation.

For each case, the following parameters were derived from the available SDP time series: number of available measurement time points; mean, median, standard deviation, variance, minimum, maximum, and range; first and last observed values; and the absolute change between the beginning and end of the observed series. The change per observation interval was additionally computed. To approximate temporal trajectory patterns, a linear slope of the SDP course was estimated; where at least three measurement points were available, a quadratic term was additionally extracted from a polynomial regression model to capture non-linear trajectories.

Features describing the temporal distribution of low amniotic fluid volumes were further derived. These included the number and proportion of measurement time points with SDP values below 1 cm or below 2 cm, the time of first and last occurrence of such low values, a weighted persistence measure emphasizing sustained late-phase oligohydramnios, and the longest consecutive series of values below 2 cm. A recovery measure was defined as the difference between the last observed value and the minimum of the series. To further contextualize the amniotic fluid situation, interaction terms were generated between minimum SDP or the proportion of low SDP values and gestational age at PPROM as well as latency duration. This approach rendered the amniotic fluid dynamics analytically accessible not as an isolated finding, but in its full clinical context.

### 2.7. Microbiological Variables and Organism Combinations

The microbiological dimension was operationalized via all variables whose designations indicated vaginal organism detection or microbiological findings at delivery. These variables underwent the same type-detection process as other predictors and were processed in numeric form where applicable. For numerically suitable microbiological variables, a presence indicator was generated, coding positive findings as 1 and absent or negative findings as 0.

On this basis, additional aggregated microbiological features were derived. For each case, the total number of positive microbiological findings was calculated. An individualized organism pattern variable was generated, combining all positive findings per case. To avoid overweighting rare individual patterns while retaining clinically relevant combination patterns, the most frequently observed patterns were explicitly coded and rarer patterns were aggregated into a residual category. This approach enabled simultaneous consideration of individual microbiological signals, overall microbial burden (expressed as the number of positive findings), and recurring combination patterns.

### 2.8. Modelling Strategy

Predictive analyses were conducted in an outcome-specific and multi-stage fashion. For binary endpoints, two model classes were applied: Elastic Net models and Random Forest models. Elastic Net modelling was chosen to enable regularized linear modelling with integrated variable selection. Random Forest models were employed to capture more complex, potentially non-linear associations. For ordinal outcomes, Random Forest models were used, with ordered outcome variables factorized for modelling purposes.

Model performance for binary outcomes was evaluated using the area under the receiver operating characteristic curve (AUC-ROC). To obtain performance estimates that are not subject to re-substitution bias, all models were evaluated using repeated 5-fold cross-validation with 10 repetitions, implemented via caret::train() with trainControl(method = ‘repeatedcv’, number = 5, repeats = 10), yielding 50 independent resampling rounds per model. In each round, the model was trained exclusively on the training partition and evaluated on the held-out validation fold, such that no observation contributed simultaneously to model training and performance estimation. Reported AUC values represent the mean across all 50 held-out evaluations. Two complementary algorithmic approaches were applied throughout: Elastic Net models (glmnet) incorporating L1/L2 regularization with integrated coefficient shrinkage and variable selection, and Random Forest models (ranger) as a structurally distinct nonlinear ensemble method. Convergence of performance estimates and variable importance patterns across these two model classes was used as an additional indicator of result robustness. Predictors were not entered unstructured into a single global model but were organized into predefined clinical domain blocks and evaluated both individually and in combination, reducing the risk of spurious associations driven by unstructured high-dimensional predictor spaces. For ordinal outcomes, repeated 5-fold cross-validation was applied with prediction accuracy as the primary performance metric. For interaction models, 5-fold cross-validation with 5 repetitions was used in consideration of model complexity. It should be noted that the exploratory risk stratification matrix (Supplementary Tables S2–S4) was derived and evaluated within the same cohort; its internal AUC values are, therefore, subject to in-sample optimism bias and are presented as descriptive plausibility estimates only, explicitly distinguished from the cross-validated ML model performance figures throughout.

All models were trained on prepared datasets containing only the variables designated for the respective outcome and model block. Observations with missing outcome data were excluded. Predictors were subsequently prepared for machine learning, imputed, and assessed for near-zero variance.

### 2.9. Multi-Stage Block Modelling

A central element of the analysis was the systematic comparison of different model configurations. First, single-block models were computed, testing each defined variable block separately against the respective outcome. This allowed the isolated predictive relevance of individual clinical information domains to be assessed.

Building on this, base-plus-block models were computed, evaluating the additional predictive contribution of each specific variable block beyond the base block alone. This step served to quantify the incremental information gain provided by additional domains when basic clinical information was already available.

In a further modelling stage, base variables, neonatal care, and ventilation were jointly modelled to represent early postnatal treatment as a clinically particularly relevant combination. Subsequently, robust overall models were computed incorporating all available predictor domains simultaneously. These overall models served to estimate the maximum predictive potential of the integrated multi-domain approach.

### 2.10. Outcome-Specific Predictor Logic

For individual outcomes, the predictor structure was selectively expanded or restricted in accordance with clinically plausible dependencies. For BPD, DLS and pulmonary hypoplasia were additionally permitted as predictors, as these variables may reflect antecedent respiratory conditions or early manifestations of pulmonary vulnerability. For the outcome death, additional neonatal complications were included in the predictor lists to allow the embedding of lethal trajectories within a complex morbidity pattern to be represented in the model. Conversely, ventilation variables were excluded from the predictor set for DLS and pulmonary hypoplasia, as these variables are more likely to represent downstream consequences of respiratory severity rather than primary input information in this context. In the context of the Model A/Model B distinction, surfactant administration—though included in the neonatal care block—is recognized as a postnatal therapeutic response variable; its appearance in model output is interpreted as mechanistic coupling rather than an antenatal predictor.

### 2.11. Interaction Analyses

To examine potential context-dependent effects of individual predictors, interaction models were additionally computed. For each model, an interaction matrix was generated from the included variables, containing both main effects and all pairwise second-order interaction terms. The interaction matrix was constructed using a model matrix without intercept; terms without variance were removed and column names were syntactically standardized for robust downstream processing.

For binary outcomes, both regularized Elastic Net models and Random Forest models were trained on this interaction matrix. For ordinal outcomes, a Random Forest approach was used. Model performance was evaluated analogously to the main models using repeated cross-validation. The primary objective of these analyses was not solely to maximize global predictive performance, but particularly to identify potentially relevant combinatorial associations among clinical, inflammatory, amniotic fluid dynamics, microbiological, and care-related features.

### 2.12. Variable Importance and Result Export

Where methodologically available, variable importance was extracted for all trained models. For Random Forest models, this was based on permutation-based importance measures. For Elastic Net models, variable contributions derived from the fitted model were documented. All results were exported in structured form, including descriptive tables, missingness summaries, performance metrics for single-block, base-plus-block, combination, overall, and interaction models, as well as corresponding variable importance rankings. This procedure established a complete and reproducible basis for subsequent result interpretation.

### 2.13. Derivation of an Exploratory Risk Stratification Matrix

Building on the overall models and variable importance analyses, an exploratory, model-derived PPROM risk matrix was developed with the aim of translating multivariate risk structures into an outcome-specific point-based scoring system suitable for analogue bedside use. Only predictor domains that had been part of the preceding modelling framework were considered. Continuous predictors—including gestational age at membrane rupture, amniotic fluid trajectory (SDP), inflammatory parameters, and latency duration—were categorized into clinically readable classes. Categorisation was guided by internal outcome-specific discriminatory performance, observed event rates, model relevance, cell count stability, and clinical interpretability.

For each category, outcome-specific score values were assigned, derived from variable importance weights, event rate differences across categories, and effect sizes. The resulting scores were summed separately per outcome, yielding an individual cumulative matrix score for each of the five endpoints: neonatal death, Dry Lung Syndrome, pulmonary hypoplasia, bronchopulmonary dysplasia, and intraventricular hemorrhage.

The internal performance of the matrix was assessed retrospectively within the study cohort using ROC analyses, AUC calculation, and exploratory cut-off evaluation. As both derivation and evaluation were conducted within the same cohort, the matrix is characterized as an exploratory, internally plausible risk stratification instrument rather than a conclusively validated clinical score. Results are presented in Supplementary Tables S2–S4.

## 3. Results

### 3.1. Cohort Characteristics and Outcome Distribution

A total of 66 pregnancies/neonates met the inclusion criteria. Maternal, perinatal, and neonatal characteristics are summarized in Table 1. Maternal age ranged from 19 to 46 years (median 34, IQR 31–37). The median gestational age at PPROM was 24 + 4 weeks (IQR 22 + 5–27 + 6), and the median duration of PPROM was 38 days (IQR 22–56). The median GA at birth was 31 + 1 weeks (IQR 27 + 3–34 + 1). Neonatal morbidity was substantial: DLS and PH each occurred in 14 cases (21.2%), neonatal death in 7 (10.6%). BPD data were available for 59 neonates; 47 (79.6%) had no BPD, and 12 (20.3%) had BPD of varying severity. IVH occurred in 9 cases (13.7%): 3 grade I and 6 grade II. Inflammation was absent in 41 cases (62.1%); infection/inflammation was documented in 19 (28.8%) and Triple I in 6 (9.1%) Results are presented in Supplementary Table S1. Pathogenic vaginal microbiome at birth was absent in 50% of cases, with single or multiple strains detected in the remainder.

### 3.2. Single-Block Model Performance and Incremental Domain Contributions

To address the distinction between antenatal counselling and postnatal prognostication, results are reported across two analytical layers. Model A comprises exclusively antenatal predictors available during expectant management—the base, inflammation, amniotic fluid dynamics (SDP), and microbiology at admission blocks. Model B encompasses the full predictor space including postnatal variables (surfactant, ventilation duration). Table 2 presents single-block AUC-ROC values for all blocks; antenatal-only block combinations are highlighted as the primary clinically actionable findings. The pattern of dominant signal sources was clearly outcome-dependent:

Death: Within Model A (antenatal predictors), the base block achieved AUC 0.962 and the combination of base + inflammation reached 0.964—exceeding the full overall model (0.958) and indicating that the antenatal prognostic signal for neonatal death is fully captured without postnatal variables. The ventilation block (Model B) achieved AUC 1.000, reflecting tautological coupling of ventilation duration with lethal outcomes and confirming reverse causation for this block.

DLS: Within Model A, the base block alone achieved AUC 0.776, and base + microbiology reached 0.775. The performance increment observed when adding the neonatal care block (base + neonatal care: 0.846) is attributable to surfactant administration—a postnatal variable reflecting therapeutic response to DLS rather than an antenatal predictor—and is therefore interpreted as a Model B mechanistic finding only. For antenatal counselling purposes, AUC 0.776 represents the available predictive signal.

PH: The base block alone (Model A) achieved AUC 0.867, and base + SDP reached 0.840—both without any postnatal variable. This represents strong antenatal predictive performance that does not require postnatal information. Surfactant appears in the top predictors of the overall model (Model B) for PH but is interpreted as a mechanistic co-variate rather than an antenatal risk factor.

BPD: No block was clearly dominant; ventilation yielded the best single-block result (RF 0.769), and overall models did not improve on this, suggesting BPD is less linearly separable in this cohort.

IVH: The base block (EN 0.824) and amniotic fluid dynamics (RF 0.806) were both informative; base + neonatal care gave the best combined result (EN 0.841).

Ordinal IVH: Consistently high accuracy (≥0.862) across all blocks—including microbiology alone (0.863)—indicates a robust and stable information structure for graded IVH outcomes.

Across outcomes, the antenatal-only block results (Model A) demonstrate that clinically meaningful predictive performance is achievable without postnatal variables for the majority of endpoints: Death (AUC 0.964), PH (AUC 0.867), IVH (AUC 0.806), and BPD (AUC 0.701). These values represent the primary counselling-relevant findings. Model B results—incorporating postnatal variables—are reported for mechanistic completeness but are not intended to inform antenatal risk stratification.

### 3.3. Integrated Overall Models, Interaction Analyses, and Regression-Based Fit

Overall models and interaction models presented in this section correspond to Model B—the full mechanistic characterization incorporating all predictor domains including postnatal variables. These results describe the complete perinatal risk trajectory and should be distinguished from the antenatal-only Model A results reported above.

**Figure 1:**
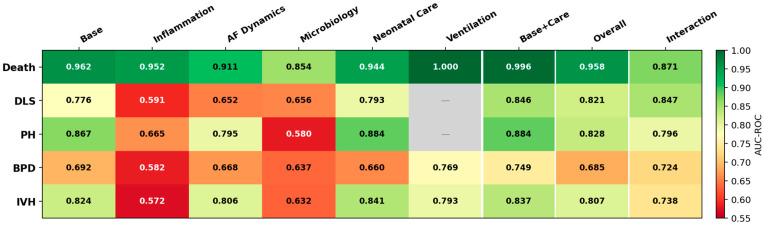
AUC-ROC heatmap across all model configurations and outcomes (best of Elastic Net/Random Forest per cell). Grey cells indicate blocks excluded by predictor logic. Base + Care = Base + Neonatal Care block (plus Ventilation for Death, BPD, IVH). AF = amniotic fluid dynamics.

Table 3 consolidates overall model performance, interaction model AUC, and complementary regression-based statistics. Figure 1 provides a visual overview of AUC-ROC across all model configurations.

Overall models confirmed multi-domain risk structure for all outcomes. For Death and DLS, performance was high (AUC 0.904–0.958 and 0.795–0.821, respectively). For PH, the overall model (RF 0.828) matched the best block-combination. BPD remained the least predictable outcome (RF 0.685), while IVH showed clear RF advantage over EN (0.807 vs. 0.705). Notably, for death, PH, and BPD, Model B (overall) performance did not exceed Model A (antenatal-only) performance, confirming that postnatal variables do not improve prediction beyond the antenatal signal for these outcomes and that their presence in the overall model reflects mechanistic rather than prospective predictive value.

Interaction models did not uniformly improve global AUC but were most informative for DLS—the Elastic Net interaction model achieved the highest AUC of any DLS model (0.847)—and provided clinically interpretable co-predictor patterns for all outcomes (Table 4).

Regression-based metrics corroborated ML findings: extended models yielded significant likelihood-ratio test improvements for Death (*p* = 0.017), DLS (*p* < 0.001), and PH (*p* < 0.001), with Nagelkerke R^2^ rising from near zero to 0.22–0.44. BPD showed no significant improvement (*p* = 0.586). Ordinal AIC improved for both BPD (ΔAIC −3.3) and IVH (ΔAIC −5.8).

### 3.4. Key Predictors by Outcome (Overall Model)

**Figure 2:**
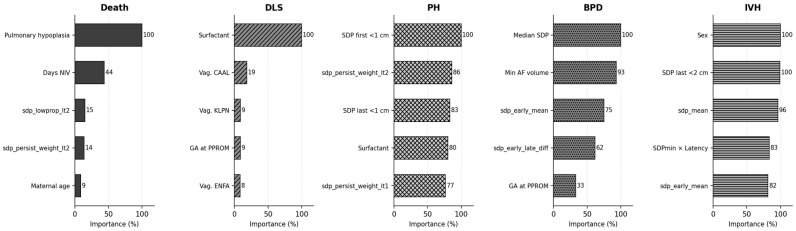
Top 5 variable importance (permutation-based, Random Forest, overall model) by outcome. Scaled relative to leading predictor per outcome (=100%). AF = amniotic fluid; GA = gestational age; NIV = non-invasive ventilation; PH = pulmonary hypoplasia; SDP = single deepest pocket.

Figure 2 shows the top five predictors from the overall Random Forest model per outcome. Distinct domain-specific predictor profiles emerged. The following variable importance profiles are derived from Model B (overall model including all predictor domains). Where postnatal variables appear as leading predictors, these reflect mechanistic coupling within the perinatal trajectory; the corresponding antenatal predictor profiles (Model A) are reported in Section 3.2:

*Death*: Pulmonary hypoplasia (co-morbidity indicator) and days of NIV dominated, alongside amniotic fluid persistence measures—reflecting a combined burden profile rather than any single predictor.

*DLS*: Surfactant administration was the leading predictor by a large margin, followed by microbiological findings and gestational age, consistent with DLS as a therapy-conditioned phenotype embedded in maturation and exposure context. As surfactant administration is a postnatal variable, this importance reflects mechanistic rather than antenatal predictive signal; the leading antenatal predictors for DLS are gestational age at PPROM and maternal age.

*PH*: All top predictors were amniotic fluid trajectory features—time of first and last SDP < 1 cm, weighted persistence of very low SDP, and surfactant—confirming that PH is captured by the temporal structure of oligohydramnios, not isolated single measurements. Surfactant, which appears in the top predictors, is a postnatal variable and is interpreted as a mechanistic downstream marker of severe respiratory disease rather than an antenatal input.

*BPD*: Median SDP, minimum amniotic fluid volume, and early-phase SDP features led, with gestational age as fifth predictor, indicating BPD as a consequence of chronic early amniotic fluid restriction combined with immaturity.

*IVH*: Sex, temporal amniotic fluid features (last SDP < 2 cm, mean SDP), and the interaction term SDPmin × Latency were top predictors, linking neurological morbidity to sustained amniotic fluid restriction in context of latency duration.

### 3.5. Interaction Analyses: Key Co-Predictor Patterns

Interaction analyses revealed consistent cross-domain risk coupling across all outcomes (Table 4). The prognostic relevance of individual variables emerged most strongly in the context of other variables: gestational maturity interacting with amniotic fluid trajectory, inflammatory status with pulmonary morbidity, and microbiological burden with respiratory therapy requirements. The risk profile following PPROM is, therefore, best characterized as a multilayered, cross-domain interaction process rather than a linear sum of independent predictor effects.

### 3.6. Supplementary Regression-Based Model Fit Statistics

To provide complementary inferential contextualization, logistic regression companion models were computed for each binary outcome, comparing a base model (sex, mode of delivery) against an extended model incorporating multi-domain predictors. For ordinal outcomes (BPD, IVH), AIC comparison between base and extended ordinal regression models was used. These analyses were not used as primary outcome anchors but serve to embed the ML findings within a classical statistical framework. Full results are presented in Table 5.

Significant likelihood-ratio test improvements were observed for Death (*p* = 0.017), DLS (*p* < 0.001), and PH (*p* < 0.001), with Nagelkerke R^2^ rising from near zero in the base model to 0.22, 0.44, and 0.31, respectively, in the extended models, and corresponding reductions in Brier Score. For BPD, no significant improvement was achieved (*p* = 0.586), consistent with the limited ML model performance for this outcome. For IVH, AUC improved from 0.608 to 0.739 but did not reach significance given the low event count (*n* = 3, *p* = 0.119). Both ordinal models showed AIC improvement in the extended specification (BPD ΔAIC −3.3; IVH ΔAIC −5.8).

### 3.7. Exploratory Risk Stratification Matrix

Building on the overall model and variable importance results, an exploratory, model-derived risk matrix was developed to translate the identified multi-domain risk structures into a clinically applicable scoring framework (Supplementary Tables S2–S4). For each of the nine clinical domain blocks—gestational age at PPROM, maternal age, vaginal microbiology at admission and at delivery, amniotic fluid trajectory, antenatal corticosteroid status, inflammatory/infectious status, gestational age at birth, and latency duration—outcome-specific scores were derived from variable importance weights, event rate differences, and effect sizes. Cumulative scores were computed per outcome and classified into four risk categories (low/moderate/elevated/high) based on empirical score quartiles from the internal cohort distribution.

Internal plausibility assessment by retrospective ROC analysis demonstrated AUC values of 0.937 for neonatal death, 0.852 for pulmonary hypoplasia, 0.843 for IVH, 0.834 for BPD, and 0.793 for DLS (Supplementary Table S4). Mean cumulative scores were consistently and substantially higher in cases with the respective outcome than in those without; for example, 11.53 vs. 7.49 for death, 7.76 vs. 5.25 for PH, and 7.79 vs. 5.68 for DLS. As derivation and internal evaluation were performed within the same cohort, these AUC values are subject to in-sample optimism and must be interpreted as exploratory plausibility estimates rather than validated predictive performance. External validation in an independent prospective cohort is required before clinical deployment.

## 4. Discussion

The present study demonstrates that neonatal outcomes following second-trimester PPROM with prolonged latency are best understood as the expression of a multilayered, cross-domain risk architecture rather than the consequence of any single dominant predictor. By integrating demographic, inflammatory, microbiological, and longitudinal amniotic fluid data within a machine learning framework, we extend our group’s prior work [10] and provide evidence that the predictive structure of each neonatal endpoint is distinctly domain-dependent. A central finding is that for the majority of outcomes—neonatal death, PH, IVH, and BPD—purely antenatal predictor blocks (Model A) achieve performance equivalent to or exceeding the full mechanistic models (Model B), confirming that the prognostic signal relevant for antenatal counselling does not depend on postnatal variables.

The central role of amniotic fluid trajectory in determining pulmonary outcomes is perhaps the most consistent and clinically interpretable finding of this study. For pulmonary hypoplasia, all top predictors in the overall model were derived from longitudinal SDP features—specifically the timing of first and last SDP < 1 cm and a weighted persistence measure for severe oligohydramnios. This is conceptually consistent with established pathophysiology: prolonged mechanical restriction of fetal thoracic expansion and impaired lung fluid dynamics during the canalicular phase of lung development (16–28 weeks) are the principal drivers of structural lung underdevelopment [7,11]. Crucially, however, our ML framework reveals that it is not any single SDP value but the temporal persistence and contextual embedding of oligohydramnios—its interaction with gestational age at rupture and latency duration—that carries the greatest predictive weight. This aligns with the observation that amniotic fluid volume at presentation is independently associated with severe neonatal respiratory morbidity and extends this insight by demonstrating that the longitudinal trajectory is more informative than the admission value alone [5,17]. Importantly, this antenatal signal is fully captured within Model A: the base block alone achieves AUC 0.867 for PH without any postnatal variable, providing a directly clinically deployable antenatal risk estimate. Critically, the absence of a group-by-time interaction in our prior longitudinal SDP analysis further supports the view that it is the sustained level—not the slope—of oligohydramnios that defines pulmonary risk [10].

The contrasting predictor profile of Dry Lung Syndrome is equally informative. DLS, defined by transient functional airway collapse following amniotic fluid loss without irreversible structural pulmonary change was dominated not by amniotic fluid dynamics but by surfactant administration, gestational parameters, and—uniquely—interaction terms between surfactant and both leucocyte maximum and gestational age [14,15,16]. This pattern suggests that DLS severity is conditioned by an intrauterinely established risk state, integrating maturity, inflammatory exposure, and therapeutic response, rather than arising as a direct consequence of oligohydramnios per se. The Elastic Net interaction model achieved the highest AUC of any DLS model (0.847, Model B); however, as this performance is driven by surfactant administration—a postnatal variable reflecting therapeutic response rather than an antenatal risk factor—it cannot be applied for prospective antenatal risk stratification. For counselling purposes, the Model A antenatal signal (base block: AUC 0.776) represents the clinically available predictive information, with gestational maturity and inflammatory status as the primary actionable risk determinants [26,27].

The microbiological dimension contributed meaningfully, particularly for neurological endpoints. Ordinal IVH was predicted with high and consistent accuracy (≥0.862) across nearly all model configurations, with amniotic fluid trajectory features and their interactions dominating variable importance. The finding that microbiological burden independently contributed to IVH risk—and interacted with SDP persistence—supports the hypothesis that sustained intrauterine inflammatory exposure, mediated in part by ascending microbial colonization, confers neurological vulnerability beyond the effects of prematurity alone [19,20,31]. The relationship between vaginal microbiome composition and intra-amniotic inflammation is well established, and the present data extend this relationship into the neonatal neurological domain [23]. Notably, the consistent predictability of ordinal IVH across blocks—including microbiology alone—suggests a robust signal that may be clinically actionable through targeted microbiome surveillance during expectant management.

For BPD, predictive performance was comparatively limited across all configurations, consistent with BPD being a complex chronic lung disease reflecting the cumulative effects of immaturity, mechanical ventilation, oxygen exposure, and intrauterine conditioning rather than any single antenatal exposure [7]. The interaction analyses pointed toward a multi-step cascade of early amniotic fluid restriction, infectious burden, and postnatal respiratory adaptation—a pattern analogous to the “double-hit” model previously proposed for BPD development in the context of chorioamnionitis and postnatal infection [7].

## 5. Limitations

Several limitations of this study require consideration. The retrospective single-centre design and modest cohort size (*n* = 66) constrain generalisability, and the low event counts for neonatal death (*n* = 7) and IVH (*n* = 9) place these endpoints near or below the events-per-variable threshold for stable model inference, even under regularization. Variable importance rankings derived from Random Forest models are susceptible to instability under conditions of event sparsity, and no formal bootstrap stability analysis was performed; findings for these outcomes should, therefore, be treated as hypothesis-generating rather than confirmatory. Results for DLS, PH, and BPD—with higher event counts—are comparatively more robust, though still subject to the general limitations of a single-centre derivation cohort. Clinical classification of DLS and PH relied on operationalised rather than histopathologically confirmed definitions, introducing a degree of diagnostic imprecision [10]. We acknowledge that mechanical ventilation duration alone does not constitute a pathological diagnosis of pulmonary hypoplasia and that overlap with severe RDS cannot be fully excluded; the classification is, therefore, interpreted as a pragmatic clinical phenotype approach suitable for retrospective analysis rather than a definitive etiologic diagnosis. Microbiological characterization was based on conventional culture methods; molecular microbiome profiling by 16S rRNA sequencing would provide substantially richer information on the vaginal microbial community [23]. The absence of direct amniotic fluid cytokine measurements (e.g., IL-6, ASC) as continuous predictors represents a gap, given their established role in intra-amniotic inflammation [19]. The primary ML model performance estimates were obtained using repeated 5-fold cross-validation with 10 repetitions, providing held-out validation performance free of re-substitution bias; however, this internal procedure does not substitute for external validation in an independent cohort, which was not feasible within the scope of this single-centre study and remains the primary objective of subsequent prospective work. The exploratory risk stratification matrix was derived and evaluated within the same cohort; its internal AUC values are subject to in-sample optimism bias and should be regarded as descriptive plausibility estimates rather than validated predictive performance metrics.

Furthermore, the full overall models (Model B) incorporate postnatal variables—specifically surfactant administration, ventilation duration, and pulmonary hypoplasia as a predictor of neonatal death—which are not available at the time of antenatal counselling and introduce reverse causation for DLS in particular; the primary clinically relevant findings are therefore the antenatal-only Model A block results, which are explicitly foregrounded throughout Section 3 and Section 4.

## 6. Future Perspectives

The findings of this study provide a proof-of-concept for multi-domain ML-based risk stratification in PPROM and define a clear translational agenda. Prospective multicentre studies incorporating standardized longitudinal SDP assessment, molecular microbiome profiling, and amniotic fluid inflammatory biomarkers are required to validate and refine the predictor structures identified here. Integration of deep learning architectures capable of modelling the full SDP time-series—rather than derived summary features—may further improve predictive granularity for PH and IVH. Clinically, a validated antenatal risk score incorporating SDP trajectory, gestational age at rupture, inflammatory status, and microbiological burden could meaningfully support individualized counselling of families navigating expectant management of second-trimester PPROM—a clinical scenario for which reliable predictive tools remain absent [8,9,10,17].

## 7. Conclusions

This study demonstrates that neonatal outcomes following second-trimester PPROM with prolonged latency are shaped by a multidimensional, cross-domain risk architecture that is not adequately captured by traditional statistical approaches. Machine learning models integrating longitudinal amniotic fluid dynamics, inflammatory status, microbiological findings, and perinatal baseline data achieved clinically meaningful predictive performance for pulmonary hypoplasia, Dry Lung Syndrome, neonatal death, and IVH, with distinct predictor profiles emerging for each endpoint. The temporal structure of oligohydramnios—rather than any single measurement—emerged as the dominant antenatal determinant of structural pulmonary morbidity, while microbiological burden contributed independently to neurological outcomes. These findings support the utility of multi-domain machine learning frameworks for individualized risk stratification in this high-risk population and provide a structured basis for prospective validation in larger, multicentre cohorts.

## Figures and Tables

**Table 1:** Demographic, maternal, and neonatal characteristics of the study cohort (*n* = 66). GA = gestational age; IQR = interquartile range; SDP = single deepest pocket; IMV = invasive mechanical ventilation; NIV = non-invasive ventilation; BPD = bronchopulmonary dysplasia; IVH = intraventricular hemorrhage; DLS = Dry Lung Syndrome; PH = pulmonary hypoplasia.

Variable	*n*	Median (IQR)	*n*/%
**Maternal characteristics**			
Maternal age (years)	66	34 (31–37)	—
GA at PPROM (week/day)	66	24 + 4 (22 + 5–27 + 6)	—
Duration of PPROM (days)	66	38 (22–56)	—
Minimum SDP (cm)	66	1.8 (1.1–2.8)	—
Median SDP (cm)	66	2.9 (2.2–4.3)	—
Maximum leucocytes (/nL)	66	13 (10–15)	—
Mode of delivery (spontaneous/C-section)	66	—	15/51 (23%/77%)
**Antenatal corticosteroids**			
None	66	—	8 (12.1%)
1 dose	66	—	0 (0%)
2 doses	66	—	55 (83.3%)
Rescue dose	66	—	3 (4.6%)
**Maternal infection/inflammation status**			
None	66	—	41 (62.1%)
Infection or inflammation	66	—	19 (28.8%)
Triple I	66	—	6 (9.1%)
**Pathogenic vaginal microbiome at birth**			
None	66	—	33 (50.0%)
Single strain	66	—	18 (27.3%)
Multiple strains	66	—	15 (22.7%)
**Neonatal characteristics**			
GA at birth (week/day)	66	31 + 1 (27 + 3–34 + 1)	—
Sex (male/female)	66	—	35/31 (53.0%/47.0%)
Surfactant administered	66	—	44 (66.7%)
IMV duration (days)	66	0 (0–4.0)	—
NIV duration (days)	66	5.0 (0–36.0)	—
**Neonatal respiratory outcomes**			
Dry Lung Syndrome (DLS)	66	—	14 (21.2%)
Pulmonary hypoplasia (PH)	66	—	14 (21.2%)
Neonatal death	66	—	7 (10.6%)
**BPD (*n* = 59 evaluable)**			
None	59	—	47 (79.6%)
Mild	59	—	8 (13.6%)
Moderate	59	—	2 (3.4%)
Severe	59	—	2 (3.4%)
**IVH (*n* = 66)**			
None	66	—	57 (86.3%)
Grade I	66	—	3 (4.6%)
Grade II	66	—	6 (9.1%)
Grade III	66	—	0 (0%)

**Table 2:** Best AUC-ROC (binary) or accuracy (ordinal) per outcome and single predictor block. Values reflect the higher of Elastic Net and Random Forest. Antenatal blocks (Model A): Base, inflammation, AF dynamics, microbiology at admission. Postnatal/peripartum blocks (Model B): Neonatal care (includes surfactant), ventilation. For antenatal counselling purposes, the Model A block combinations represent the clinically deployable predictive signal. * = best block per row. - = excluded by predictor logic (ventilation variables withheld for DLS/PH). AF = amniotic fluid dynamics block.

Outcome	Base	Inflammation	AF Dynamics	Microbiology	Neonatal Care	Ventilation
Death	0.962	0.952	0.911	0.854	0.944	1.000 *
DLS	0.776	0.591	0.652	0.656	0.793 *	-
PH	0.867	0.665	0.795	0.580	0.884 *	-
BPD	0.692	0.582	0.668	0.637	0.660	0.769 *
IVH	0.824	0.572	0.806	0.632	0.841 *	0.793
BPD (ord.) Accuracy	0.546	0.516	0.515	0.597	0.572	0.613 *
IVH (ord.) Accuracy	0.839	0.829	0.862	0.863 *	0.863	0.834

**Table 3:** Model performance summary across overall models, interaction models, and regression-based fit statistics. EN = Elastic Net; RF = Random Forest; ext. = extended model including multi-domain predictors; * = highest AUC for that outcome across all models; Ordinal outcomes reported as accuracy; AIC comparison for ordinal regression. Regression AUC and Nagelkerke R^2^ from logistic companion models.

Outcome	Overall EN	Overall RF	Interact. EN	Interact. RF	Regr. AUC (Base → ext.)	Nagelkerke R^2^ (ext.)	Model Compar.
Death	0.958	0.904	0.842	0.871	0.637 → 0.766	0.223	0.017
DLS	0.795	0.821	0.847 *	0.795	0.524 → 0.762	0.444	<0.001
PH	0.824	0.828	0.796	0.778	0.610 → 0.772	0.313	<0.001
BPD	0.632	0.685	0.649	0.724	0.525 → 0.549	0.010	0.586
IVH	0.705	0.807	0.589	0.738	0.608 → 0.739	0.143	0.119
BPD (ord.) Accuracy	—	0.591	—	0.597	AIC: 153 → 150	—	ΔAIC −3.3
IVH (ord.) Accuracy	—	0.863	—	0.862	AIC: 65.3 → 59.4	—	ΔAIC −5.8

**Table 4:** Key interaction patterns by outcome. Interactions listed by relative importance weight from Elastic Net and Random Forest interaction models. AF = amniotic fluid; GA = gestational age; NIV = non-invasive ventilation; PH = pulmonary hypoplasia; SDP = single deepest pocket.

Outcome	Key Interactions (Highest Weights)	Clinical Interpretation
Death	Latency × PH; Leucocytes max × PH; Sex × Days NIV	Lethal outcomes driven by coupling of pulmonary impairment + inflammatory activity + respiratory support burden
DLS	GA at PPROM × Surfactant; Maternal age × Surfactant; Leucocytes max × Surfactant; Median SDP × Surfactant	DLS severity conditioned by maturity and inflammatory state—therapy need signals intrauterine risk
PH	Leucocytes × SDPmin × GA; GA at PPROM × SDP persistence; Latency × First SDP < 2 cm	PH linked to persistence and timing of amniotic fluid restriction, not single measurements
BPD	Microbiology × Surfactant; Late AF phase × Microbiology; Latency × Early AF pattern	BPD as cascade of intrauterine burden, infectious status and postnatal respiratory adaptation
IVH	Median SDP × First SDP < 1 cm; GA birth × Mean SDP; sdp_lowcount_lt2 × Microbiology	Neurological vulnerability structured by AF dynamics interacting with maturity and microbiology

**Table 5:** Supplementary regression-based model fit statistics. Binary outcomes: logistic regression base model (sex, mode of delivery) vs. extended multi-domain model; likelihood-ratio test (LRT). Ordinal outcomes: AIC of base vs. extended ordinal regression. AUC = area under ROC curve; R^2^ = Nagelkerke R^2^; BS = Brier Score; LRT *p* = likelihood-ratio test *p*-value; ΔAIC = AIC extended − AIC base.

Outcome	*n*	Events	AUC/AIC (Base)	AUC/AIC (ext.)	Nagelkerke R^2^/ΔAIC	Brier Score (Base)	Brier Score (ext.)	LRT *p*
**Binary outcomes (logistic regression)**								
Death	66	7	0.637	0.766	0.223	0.092	0.086	**0.017**
DLS	66	24	0.524	0.762	0.444	0.231	0.165	**<0.001**
PH	66	14	0.610	0.772	0.313	0.162	0.139	**<0.001**
BPD	59	38	0.525	0.549	0.010	0.241	0.240	0.586
IVH	66	3	0.608	0.739	0.143	0.044	0.043	0.119
**Ordinal outcomes (ordinal regression—AIC)**								
BPD (ordinal)	59	—	153.2	149.9	−3.3	—	—	—

## Data Availability

In the context of self-research, recording as well as storage of all personal data already known through treatment in pseudonymized form in a database in the network of the clinic for 10 years (§ 630f BGB). No passing on of data to third parties. No data will be passed on to persons who are not part of the study team. Guarantee of data security by exclusive use of the clinic network as well as securing the database by means of a password known only to the study team. Publication of data exclusively in anonymized form. The datasets generated during and/or analyzed during the current study are available from the corresponding author on reasonable request.

## Supplementary Materials

The following supporting information can be downloaded at: https://www.mdpi.com/article/10.3390/diagnostics16121911/s1, Table S1: Distribution of neonatal respiratory outcomes according to maternal inflammatory status; Table S2a: Domain-specific risk scores per outcome; Table S2b: Observed event rates per domain category; Table S3: Cumulative risk score thresholds by outcome; Table S4: Internal validation performance.
