# Supplementary material for: Maternal and Neonatal Determinants of Respiratory Outcome Following Second-Trimester PPROM: A Multi-Domain Machine Learning Analysis
Source: Diagnostics (Basel). 2026 Jun 19;16(12):1911. doi: 10.3390/diagnostics16121911 (PMC13298293; doi:10.3390/diagnostics16121911)
Supplement: Supplementary file 1 [file diagnostics-16-01911-s001.zip › diagnostics-4341436 Paper_PROMISE_ML_Supplement MultiMatrix.pdf]

Supplementary Material — PROMISE: Multi-Domain Risk Scoring Matrix

Supplementary Tables S2–S4. Exploratory, model-derived risk stratification matrix for neonatal outcome prediction following second-trimester PPROM with prolonged latency. Scores are derived from variable importance weights, outcome-specific event rate differences, and effect sizes extracted from the Elastic Net and Random Forest overall models. The matrix is designed as an analogue reference tool for rapid bedside risk assessment, structured analogously to a ward-based scoring system.

Methodological basis of score derivation

Score values for each domain category were derived exclusively from predictor domains that formed part of the preceding multi-domain machine learning analysis. Continuous predictors (gestational age, amniotic fluid trajectory, inflammatory parameters, latency) were categorised into clinically readable classes. Categorisation was guided by internal outcome-specific discriminatory performance, observed event rates, model-derived variable importance, cell count stability, and clinical interpretability. Outcome-specific scores were assigned based on variable importance weights, event rate differences across categories, and effect sizes, and were smoothed empirically where cell counts were small. Scores for each domain are summed per outcome to generate a cumulative risk score, which is then classified using the risk thresholds in Table S3 (empirical quartiles from the internal cohort distribution).

Important note on the corticosteroid domain: the corticosteroid domain was clinically re-coded. Model-derived weights were not applied directly, as the data-driven direction was considered clinically counterintuitive. Complete documentation of an antenatal corticosteroid course was assigned 0 points (reference); incomplete, absent, or unclear documentation was assigned risk points.

Score colour coding legend (statistically grounded)

(white)	Score = 0 — reference category (no additional risk contribution for this domain)
(green)	Score > 0 and below within-outcome mean of non-zero scores — below-average risk contribution
(yellow)	Score ≥ within-outcome mean of non-zero scores — above-average risk contribution
(pink)	Score ≥ 2.0 — high risk contribution (corresponds to approximately ≥ 1 SD above mean for most outcomes)

Within-outcome means of non-zero scores used for colour classification: Death 1.10, DLS 0.96, PH 0.98, BPD 0.84, IVH 0.94.

* Very small cell count (n < 5): scores should be interpreted with particular caution.

† Small cell count (n = 5–10): scores are exploratory; confidence intervals would be wide.

Supplementary Table S2a — Domain-specific risk scores per outcome

For each domain block (A–I), select the single criterion that best matches the patient profile. The corresponding outcome-specific score is added to the cumulative risk score for that outcome. Select the most severe applicable category within each domain.

Domain	Category	Criterion / Selection rule	Stability	n	Death	DLS	PH	BPD	IVH
**A | Gestational age at PPROM**									
GA at PPROM	18+0–18+6	PPROM between 18+0 and 18+6 weeks	No internal cases	—	1.6	1.0	1.3	0.1	1.4
	19+0–19+6	PPROM between 19+0 and 19+6 weeks	Very small cell count*	4	1.4	0.9	1.2	0.1	1.3

Domain	Category	Criterion / Selection rule	Stability	n	Death	DLS	PH	BPD	IVH
	20+0–20+6	PPROM between 20+0 and 20+6 weeks	Very small cell count*	2	1.3	0.9	1.1	0.1	1.2
	21+0–21+6	PPROM between 21+0 and 21+6 weeks	Small cell count†	5	1.2	0.8	1.0	0.1	1.1
	22+0–22+6	PPROM between 22+0 and 22+6 weeks	Very small cell count*	4	1.1	0.7	0.9	0.1	1.0
	23+0–23+6	PPROM between 23+0 and 23+6 weeks	Adequate for exploratory use	13	1.0	0.6	0.8	0	0.9
	24+0–24+6	PPROM between 24+0 and 24+6 weeks	Very small cell count*	4	0.9	0.6	0.8	0	0.8
	25+0–25+6	PPROM between 25+0 and 25+6 weeks	Very small cell count*	4	0.8	0.5	0.7	0	0.7
	26+0–26+6	PPROM between 26+0 and 26+6 weeks	Small cell count†	6	0.7	0.4	0.6	0	0.6
	27+0–27+6	PPROM between 27+0 and 27+6 weeks	Small cell count†	6	0.6	0.4	0.5	0	0.5
	28+0–28+6	PPROM between 28+0 and 28+6 weeks	Very small cell count*	3	0.4	0.3	0.4	0	0.4
	29+0–29+6	PPROM between 29+0 and 29+6 weeks	Very small cell count*	3	0.3	0.2	0.3	0	0.3
	30+0–30+6	PPROM between 30+0 and 30+6 weeks	Small cell count†	5	0.2	0.1	0.2	0	0.2
	31+0–31+6	PPROM between 31+0 and 31+6 weeks	Small cell count†	5	0.1	0.1	0.1	0	0.1
	32+0–32+6	PPROM between 32+0 and 32+6 weeks (reference)	No internal cases	—	0	0	0	0	0
**B | Maternal age**									
Maternal age	< 25 yrs	Maternal age < 25 years	Very small cell count*	4	1.5	0.9	1.5	1.5	0.2
	25–29 yrs	Maternal age 25 to 29 years	Small cell count†	8	0.4	1.5	0.9	0.4	1.5
	30–34 yrs	Maternal age 30 to 34 years	Adequate for exploratory use	28	0.3	0.5	0.3	0.6	0.3
	35–39 yrs	Maternal age 35 to 39 years	Adequate for exploratory use	20	0	0	0	0.8	0.6

Domain	Category	Criterion / Selection rule	Stability	n	Death	DLS	PH	BPD	IVH
	≥ 40 yrs	Maternal age ≥ 40 years	Small cell count†	6	0.1	0.4	0.3	0	0
**C | Vaginal microbiology at admission**									
Microbiology at admission	None	No clinically relevant pathogen at admission	Adequate	11	0	0	0	0	0
	1 pathogen	Exactly one clinically relevant pathogen at admission	Small cell count†	7	1.9	0.6	0	0.2	0.5
	≥ 2 pathogens	Two or more clinically relevant pathogens at admission	Adequate	48	1.9	1.2	0.2	0.2	2.0
**D | Amniotic fluid trajectory (serial SDP measurements)**									
AF trajectory (SDP)	No oligohydramnios	SDP predominantly > 3.0 cm; no oligo-/anhydramnios	Adequate	15	0	0	0	0	0
	Borderline	Intermittent values ≤ 3.0 cm; no severe/persistent pattern	Small cell count†	6	0.6	0.7	0.6	2.1	0.6
	Low / unfavourable	At least one SDP ≤ 2.0 cm or predominantly borderline	Adequate	12	1.5	1.8	2.6	2.1	0.6
	Severe/persistent	Repeated SDP ≤ 2.0 cm or ≥ 75% of measurements ≤ 3.0 cm	Adequate	33	2.8	1.8	3.0	2.4	1.2
**E | Antenatal corticosteroid therapy (lung maturation)**									
Antenatal corticosteroids	Complete	Complete antenatal corticosteroid course documented (reference)	Adequate	58	0	0	0	0	0
	Incomplete/unclear	Incomplete, absent, or documentation unclear	Small cell count†	8	1.0	2.5	2.0	0.9	1.4
**F | Maternal inflammatory / infectious status**									
Inflammation / Infection	None	No relevant CRP/leucocyte signal; no documented infection	Adequate	15	0	0	0	0	0
	Elevated	CRP ≥ 10 mg/l or leucocytes ≥ 15/nl or clinical signs of infection	Adequate	12	2.5	1.1	1.3	0	0.8
	Markedly elevated	CRP ≥ 17 mg/l or documented AIS / chorioamnionitis / Triple I	Adequate	39	2.5	1.1	1.6	0	0.8

Domain	Category	Criterion / Selection rule	Stability	n	Death	DLS	PH	BPD	IVH
**G | Vaginal microbiology at delivery**									
Microbiology at delivery	None	No clinically relevant pathogen at delivery (reference)	Adequate	33	0	0	0	0	0
	1 pathogen	Exactly one clinically relevant pathogen at delivery	Adequate	18	0	1.1	0	0.3	0.8
	≥ 2 pathogens	Two or more clinically relevant pathogens at delivery	Adequate	15	1.0	1.1	0	0.4	1.0
**H | Gestational age at birth**									
GA at birth	24+0–24+6	Delivery between 24+0 and 24+6 weeks	Very small cell count*	3	2.0	2.0	2.0	1.5	2.0
	25+0–25+6	Delivery between 25+0 and 25+6 weeks	Small cell count†	5	2.0	2.0	1.4	1.5	2.0
	26+0–26+6	Delivery between 26+0 and 26+6 weeks	Small cell count†	5	1.5	2.0	1.4	1.3	2.0
	27+0–27+6	Delivery between 27+0 and 27+6 weeks	Small cell count†	6	1.5	2.0	1.2	1.3	1.8
	28+0–28+6	Delivery between 28+0 and 28+6 weeks	Very small cell count*	4	1.1	2.0	1.1	1.3	1.8
	29+0–29+6	Delivery between 29+0 and 29+6 weeks	Small cell count†	6	1.1	2.0	1.1	0.8	1.1
	30+0–30+6	Delivery between 30+0 and 30+6 weeks	Very small cell count*	2	1.1	2.0	1.1	0.8	0.9
	31+0–31+6	Delivery between 31+0 and 31+6 weeks	Small cell count†	6	0.5	0.2	0.5	0.8	0.9
	32+0–32+6	Delivery between 32+0 and 32+6 weeks	Very small cell count*	2	0.5	0.2	0.4	0.8	0.3
	33+0–33+6	Delivery between 33+0 and 33+6 weeks	Small cell count†	7	0.5	0.2	0.4	0.8	0
	34+0–34+6	Delivery between 34+0 and 34+6 weeks	Small cell count†	9	0	0.2	0	0.8	0
	35+0–35+6	Delivery between 35+0 and 35+6 weeks	Very small cell count*	2	0	0.2	0	0.8	0
	36+0 and above	Delivery ≥ 36+0 weeks (reference)	Small cell count†	8	0	0	0	0	0

Domain	Category	Criterion / Selection rule	Stability	n	Death	DLS	PH	BPD	IVH
Latency period	< 14 days	Latency < 14 days (not represented internally)	No internal cases	—	0	0	0	0	0
	14–27 days	Latency 14 to 27 days (reference)	Adequate	22	0	0	0	0.1	0
**I | Latency period (PPROM to delivery)**									
	28–41 days	Latency 28 to 41 days	Adequate	15	0.3	1.2	0.8	0.8	1.0
	42–55 days	Latency 42 to 55 days	Adequate	12	1.5	1.0	1.2	0.3	0.8
	≥ 56 days	Latency ≥ 56 days	Adequate	17	0.6	0.3	0.3	0	0.3

Supplementary Table S2b — Observed event rates per domain category (internal cohort, n = 66)

Observed event frequencies and crude event rates within each domain category from the internal derivation cohort. These rates served as one component of score derivation and are presented here for transparency. ev = events; n = evaluable cases in category; % = crude event rate. Due to small cell counts in several categories, rates should be interpreted as descriptive only.

Domain	Category	Death (ev/n, %)	DLS (ev/n, %)	PH (ev/n, %)	BPD (ev/n, %)	IVH (ev/n, %)
**A | Gestational age at PPROM**						
GA at PPROM	18+0–18+6	—	—	—	—	—
	19+0–19+6	1/4 (25.0%)	3/4 (75.0%)	1/4 (25.0%)	3/3 (100.0%)	2/4 (50.0%)
	20+0–20+6	2/2 (100.0%)	2/2 (100.0%)	2/2 (100.0%)	2/2 (100.0%)	1/2 (50.0%)
	21+0–21+6	1/5 (20.0%)	4/5 (80.0%)	4/5 (80.0%)	5/5 (100.0%)	1/5 (20.0%)
	22+0–22+6	0/4 (0.0%)	1/4 (25.0%)	0/4 (0.0%)	4/4 (100.0%)	1/4 (25.0%)
	23+0–23+6	1/13 (7.7%)	7/13 (53.8%)	4/13 (30.8%)	12/13 (92.3%)	1/13 (7.7%)
	24+0–24+6	0/4 (0.0%)	0/4 (0.0%)	0/4 (0.0%)	3/4 (75.0%)	1/4 (25.0%)
	25+0–25+6	0/4 (0.0%)	0/4 (0.0%)	1/4 (25.0%)	2/4 (50.0%)	0/4 (0.0%)
	26+0–26+6	1/6 (16.7%)	2/6 (33.3%)	1/6 (16.7%)	3/5 (60.0%)	0/5 (0.0%)
	27+0–27+6	0/6 (0.0%)	3/6 (50.0%)	0/6 (0.0%)	4/6 (66.7%)	1/6 (16.7%)
	28+0–28+6	0/3 (0.0%)	0/3 (0.0%)	0/3 (0.0%)	2/3 (66.7%)	0/3 (0.0%)

Domain	Category	Death (ev/n, %)	DLS (ev/n, %)	PH (ev/n, %)	BPD (ev/n, %)	IVH (ev/n, %)
	29+0–29+6	0/3 (0.0%)	0/3 (0.0%)	0/3 (0.0%)	2/3 (66.7%)	0/3 (0.0%)
	30+0–30+6	0/5 (0.0%)	1/5 (20.0%)	0/5 (0.0%)	3/5 (60.0%)	0/5 (0.0%)
	31+0–31+6	0/5 (0.0%)	0/5 (0.0%)	0/5 (0.0%)	3/5 (60.0%)	0/5 (0.0%)
	32+0–32+6	—	—	—	—	—
**B | Maternal age**						
Maternal age	< 25 yrs	3/4 (75.0%)	2/4 (50.0%)	3/4 (75.0%)	3/3 (100.0%)	0/3 (0.0%)
	25–29 yrs	1/8 (12.5%)	5/8 (62.5%)	3/8 (37.5%)	6/8 (75.0%)	3/8 (37.5%)
	30–34 yrs	3/28 (10.7%)	10/28 (35.7%)	5/28 (17.9%)	21/27 (77.8%)	3/28 (10.7%)
	35–39 yrs	0/20 (0.0%)	5/20 (25.0%)	2/20 (10.0%)	16/20 (80.0%)	3/20 (15.0%)
	≥ 40 yrs	0/6 (0.0%)	2/6 (33.3%)	1/6 (16.7%)	4/6 (66.7%)	0/6 (0.0%)
**C | Vaginal microbiology at admission**						
Microbiology at admission	None	0/11 (0.0%)	2/11 (18.2%)	2/11 (18.2%)	9/11 (81.8%)	0/11 (0.0%)
	1 pathogen	1/7 (14.3%)	2/7 (28.6%)	1/7 (14.3%)	6/7 (85.7%)	0/7 (0.0%)
	≥ 2 pathogens	6/48 (12.5%)	20/48 (41.7%)	11/48 (22.9%)	35/46 (76.1%)	9/47 (19.1%)
**D | Amniotic fluid trajectory (serial SDP measurements)**						
AF trajectory (SDP)	No oligohydramnios	0/15 (0.0%)	2/15 (13.3%)	0/15 (0.0%)	7/15 (46.7%)	1/15 (6.7%)
	Borderline	0/6 (0.0%)	2/6 (33.3%)	0/6 (0.0%)	5/6 (83.3%)	0/6 (0.0%)
	Low / unfavourable	2/12 (16.7%)	6/12 (50.0%)	3/12 (25.0%)	11/12 (91.7%)	2/12 (16.7%)
	Severe/persistent	5/33 (15.2%)	14/33 (42.4%)	11/33 (33.3%)	27/31 (87.1%)	6/32 (18.8%)
**E | Antenatal corticosteroid therapy (lung maturation)**						
Antenatal corticosteroids	Complete	7/58 (12.1%)	24/58 (41.4%)	14/58 (24.1%)	45/56 (80.4%)	9/57 (15.8%)

Domain	Category	Death (ev/n, %)	DLS (ev/n, %)	PH (ev/n, %)	BPD (ev/n, %)	IVH (ev/n, %)
	Incomplete/unclear	0/8 (0.0%)	0/8 (0.0%)	0/8 (0.0%)	5/8 (62.5%)	0/8 (0.0%)
**F | Maternal inflammatory / infectious status**						
Inflammation / Infection	None	0/15 (0.0%)	4/15 (26.7%)	2/15 (13.3%)	12/15 (80.0%)	2/15 (13.3%)
	Elevated	1/12 (8.3%)	3/12 (25.0%)	2/12 (16.7%)	9/12 (75.0%)	0/12 (0.0%)
	Markedly elevated	6/39 (15.4%)	17/39 (43.6%)	10/39 (25.6%)	29/37 (78.4%)	7/38 (18.4%)
**G | Vaginal microbiology at delivery**						
Microbiology at delivery	None	3/33 (9.1%)	9/33 (27.3%)	9/33 (27.3%)	24/32 (75.0%)	3/32 (9.4%)
	1 pathogen	1/18 (5.6%)	9/18 (50.0%)	3/18 (16.7%)	14/18 (77.8%)	3/18 (16.7%)
	≥ 2 pathogens	3/15 (20.0%)	6/15 (40.0%)	2/15 (13.3%)	12/14 (85.7%)	3/15 (20.0%)
**H | Gestational age at birth**						
GA at birth	24+0–24+6	0/3 (0.0%)	2/3 (66.7%)	2/3 (66.7%)	3/3 (100.0%)	0/3 (0.0%)
	25+0–25+6	2/5 (40.0%)	3/5 (60.0%)	2/5 (40.0%)	5/5 (100.0%)	2/5 (40.0%)
	26+0–26+6	0/5 (0.0%)	3/5 (60.0%)	2/5 (40.0%)	4/5 (80.0%)	2/5 (40.0%)
	27+0–27+6	2/6 (33.3%)	3/6 (50.0%)	2/6 (33.3%)	5/5 (100.0%)	1/6 (16.7%)
	28+0–28+6	0/4 (0.0%)	2/4 (50.0%)	1/4 (25.0%)	4/4 (100.0%)	2/4 (50.0%)
	29+0–29+6	1/6 (16.7%)	4/6 (66.7%)	2/6 (33.3%)	4/6 (66.7%)	1/6 (16.7%)
	30+0–30+6	1/2 (50.0%)	2/2 (100.0%)	1/2 (50.0%)	2/2 (100.0%)	0/2 (0.0%)
	31+0–31+6	0/6 (0.0%)	1/6 (16.7%)	1/6 (16.7%)	5/6 (83.3%)	1/6 (16.7%)
	32+0–32+6	0/2 (0.0%)	0/2 (0.0%)	0/2 (0.0%)	0/2 (0.0%)	0/2 (0.0%)
	33+0–33+6	1/7 (14.3%)	1/7 (14.3%)	1/7 (14.3%)	4/6 (66.7%)	0/6 (0.0%)
	34+0–34+6	0/9 (0.0%)	2/9 (22.2%)	0/9 (0.0%)	8/9 (88.9%)	0/9 (0.0%)

Domain	Category	Death (ev/n, %)	DLS (ev/n, %)	PH (ev/n, %)	BPD (ev/n, %)	IVH (ev/n, %)
	35+0–35+6	0/2 (0.0%)	1/2 (50.0%)	0/2 (0.0%)	2/2 (100.0%)	0/2 (0.0%)
	36+0 and above	0/8 (0.0%)	0/8 (0.0%)	0/8 (0.0%)	3/8 (37.5%)	0/8 (0.0%)
Latency period	< 14 days	—	—	—	—	—
	14–27 days	0/22 (0.0%)	5/22 (22.7%)	3/22 (13.6%)	16/22 (72.7%)	2/22 (9.1%)
**I | Latency period (PPROM to delivery)**						
	28–41 days	1/15 (6.7%)	8/15 (53.3%)	4/15 (26.7%)	14/15 (93.3%)	3/15 (20.0%)
	42–55 days	4/12 (33.3%)	6/12 (50.0%)	4/12 (33.3%)	8/10 (80.0%)	2/11 (18.2%)
	≥ 56 days	2/17 (11.8%)	5/17 (29.4%)	3/17 (17.6%)	12/17 (70.6%)	2/17 (11.8%)

Supplementary Table S3 — Cumulative risk score thresholds by outcome

Risk categories are based on empirical score quartiles from the internal derivation cohort. Thresholds are exploratory and require external validation before clinical application. Low / moderate / elevated / high risk categories are colour-coded consistently with the variable importance legend in Table S2a.

Outcome	Low risk	Moderate risk	Elevated risk	High risk
**Neonatal death**	≤ 6.1	> 6.1 to 8.2	> 8.2 to 9.8	> 9.8
**Dry Lung Syndrome (DLS)**	≤ 5.2	> 5.2 to 6.7	> 6.7 to 8.0	> 8.0
**Pulmonary hypoplasia (PH)**	≤ 4.0	> 4.0 to 6.3	> 6.3 to 7.5	> 7.5
**Bronchopulmonary dysplasia (BPD)**	≤ 3.6	> 3.6 to 4.3	> 4.3 to 5.0	> 5.0
**Intraventricular haemorrhage (IVH)**	≤ 5.0	> 5.0 to 6.2	> 6.2 to 7.1	> 7.1

Supplementary Table S4 — Internal validation performance

Internal AUC-ROC values were obtained by applying the matrix scores retrospectively to the same derivation cohort (n = 66). These values reflect internal plausibility only and are subject to optimism bias, as derivation and evaluation were performed in the same dataset. External validation in an independent cohort is required before any clinical application. Mean scores are presented separately for cases with and without each outcome to illustrate score discrimination.

Outcome	n	Events	Internal AUC	Mean score (outcome present)	Mean score (outcome absent)	Note
**Neonatal death**	66	7	**0.937**	11.53	7.49	*Internal plausibility check only; derivation and evaluation performed in the same cohort (n=66). External validation required.*
**Dry Lung Syndrome (DLS)**	66	24	**0.793**	7.79	5.68	*Internal plausibility check only; derivation and evaluation performed in the same cohort (n=66). External validation required.*
**Pulmonary hypoplasia (PH)**	66	14	**0.852**	7.76	5.25	*Internal plausibility check only; derivation and evaluation performed in the same cohort (n=66). External validation required.*
**Bronchopulmonary dysplasia (BPD)**	64	50	**0.834**	4.36	2.73	*Internal plausibility check only; derivation and evaluation performed in the same cohort (n=66). External validation required.*
**Intraventricular haemorrhage (IVH)**	65	9	**0.843**	8.14	5.69	*Internal plausibility check only; derivation and evaluation performed in the same cohort (n=66). External validation required.*

Instructions for use

1. For each of the nine domain blocks (A–I), identify the single criterion that best matches the patient's clinical profile and note the corresponding score for the outcome of interest.
2. Where multiple criteria within a domain could apply (e.g. SDP trajectory, inflammation), select the most severe applicable category.
3. Sum the nine domain scores to obtain the cumulative risk score for that outcome.
4. Locate the cumulative score in Table S3 to assign the risk category (low / moderate / elevated / high).
5. Repeat steps 1–4 independently for each outcome (Death, DLS, PH, BPD, IVH).
6. The resulting multi-outcome risk profile may support individualised antenatal counselling and shared decision-making in pregnancies complicated by second-trimester PPROM with prolonged latency.

Caution: This matrix is exploratory and derived from a retrospective single-centre cohort of 66 cases. It has not been externally validated. Internal AUC values are likely optimistic due to in-sample evaluation. The matrix is intended to supplement — not replace — clinical judgement and established interdisciplinary management guidelines. Score point values and risk thresholds require prospective validation in an independent cohort before any clinical deployment.
