# Supplementary material for: Maternal and Neonatal Determinants of Respiratory Outcome Following Second-Trimester PPROM: A Multi-Domain Machine Learning Analysis
Source: Diagnostics (Basel). 2026 Jun 19;16(12):1911. doi: 10.3390/diagnostics16121911 (PMC13298293; doi:10.3390/diagnostics16121911)
Supplement: Supplementary file 1 [file diagnostics-16-01911-s001.zip › diagnostics-4341436 Paper_PROMISE_ML_Supplement.pdf]

Supplemental material:

Supplemental Table S1: Distribution of neonatal respiratory outcomes according to maternal inflammatory status. Frequencies of dry lung (DL), pulmonary hypoplasia (PH), neonatal death, and bronchopulmonary dysplasia (BPD) are presented across three maternal categories: no inflammation, Infection/inflammation, and Triple I. Outcomes are reported as absolute counts within each inflammatory group. Respiratory diagnoses (DL and PH) were mutually exclusive; death and BPD could co-occur with respiratory outcomes.

	None (n=38/66) n= / (%)	Dry Lung (n=14/66) n= / (%)	Pulmonary Hypoplasia (n=14/66) n= / %	Death (n=7/66) n= / %	BPD (n=12/59) n= / %
None (n=41/66)	25	8	8	3	9
Infection / Inflammation (n=19/66)	8	3	5	3	2
Triple I (n=6/66)	8	3	1	1	1
